# Removal of Inorganic Salts in Municipal Solid Waste Incineration Fly Ash Using a Washing Ejector and Its Application for CO_2_ Capture

**DOI:** 10.3390/ijerph19042306

**Published:** 2022-02-17

**Authors:** Hyunsoo Kim, Oyunbileg Purev, Kanghee Cho, Nagchoul Choi, Jaewon Lee, Seongjin Yoon

**Affiliations:** 1 Department of Energy and Resource Engineering, Chosun University, Gwangju 61452, Korea; star8538@naver.com (H.K.); oyunbileg@chosun.kr (O.P.); 2 Research Institute of Agriculture and Life Sciences, Seoul National University, Seoul 08826, Korea; nagchoul@snu.ac.kr; 3 JIU Corporation, Seoul 07528, Korea; jaewlee@jiuene.com; 4 Daewoong Corporation, Yeosu-si 59601, Korea; jiniyoon9@gmail.com

**Keywords:** fly ash, cavitation bubble, washing ejector, inorganic salts, CO_2_ capture

## Abstract

This study investigated the effects of washing equipment for inorganic salts, such as NaCl, KCl, and CaClOH, to decontaminate municipal solid waste incineration fly ash (MSW-IFA). Based on the feature of hydrodynamic cavitation, the device developed in this study (referred to as a ‘washing ejector’) utilizes the cavitation bubbles. A washing ejector was analyzed under a range of conditions, employing as little water as possible. In hydrodynamic cavitation, the increase in fluid pressure with increasing static pressure is mainly attributed to the increase in particle–bubble collisions via the cavitation flow. The results revealed that the fluid pressure influenced the removal of inorganic salts during cavitation in water. This is because during the washing process from the collapse of cavitation bubbles, the release is achieved through the dissolution of inorganic salts weakly bound to the surface. After treatment by a washing ejector, the removal of soluble salts elements such as Cl, Na, and K was reduced by approximately 90%. Removing the inorganic salts in the IFA altered the characteristics of the Ca-related phase, and amorphous CaCO_3_ was formed as the cavitation flow reacted with CO_2_ in the ambient air. Furthermore, the washing effluent produced by washing IFA was found to be beneficial for CO_2_ capture. The washing effluent was enriched with dissolved Ca from the IFA, and the initial pH was the most favorable condition for the formation of CaCO_3_; thus, the effluent was sufficient for use as a CO_2_ sequestration medium and substitute for the reuse of water. Overall, the process presented herein could be effective for removing soluble salts from IFA, and this process is conducive to utilizing IFA as a resource.

## 1. Introduction

Energy consumption has dramatically increased over the last three decades owing to rapid urbanization and industrialization. In particular, the enormous consumption of fossil resources is considered a major contributor to the continually increasing carbon dioxide (CO_2_) emissions [1,2]. Climate change, including global warming driven by human-induced greenhouse gas emissions, has become a significant environmental problem worldwide. This is further aggravated by thermal power generation, which creates residual materials and particulate pollution in the atmosphere [3,4]. As such, reducing CO_2_ emissions via an appropriate energy recovery process, such as waste-to-energy projects, offers favorable prospects for minimizing energy problems [5,6]. Waste-to-energy projects are widely applied as efficient methods of managing municipal solid waste (MSW). With the increase in MSW, incineration based on the concept of waste-to-energy is a commonly accepted solution in society, mainly because of the advantages of a shorter processing cycle, reduced space demands, high mass/volume reduction (overall, 70–90% of MSW), and its help in comprehensively utilizing resources [7,8]. Furthermore, the demand for daily waste disposal continues to increase owing to the rapid expansion of urbanization. However, waste incineration may produce harmful by-products [9,10], such as combustion residues after incineration; these are classified into MSW incineration fly ash (IFA) and MSW incineration bottom ash (IBA). IFA is the residual material collected in the dust collector after combustion, and IBA is the residue deposited at the sidewall and bottom of the incinerator after combustion [11,12].

Currently, combustion residues are recycled as materials for construction [13] and ceramics [14]; however, recycling IFA is relatively limited because of the presence of hazardous elements, such as toxic metals (Cd, Cu, Hg, Pb, and Zn) and dioxins (PCDD/Fs). In particular, it has been reported that chlorine derived from food waste and PVC in the MSW forms metal chloride in the IFA, resulting in the chemical partitioning of metals facilitated by the presence of chlorine [15,16]. Hence, the high content of inorganic salts is likely to prevent the recyclability of IFA. To date, the pre-treatment techniques for hazardous IFA have been achieved by using leaching methods such as water leaching [17], CO_2_-water leaching [18,19], and various acid-based leaching methods [20]. Among these treatment techniques, many researchers have utilized water washing to achieve a high chlorine removal rate from IFA to remove significant amounts of inorganic salts. Many investigations report an applied pre-treatment using single or multiple treatment methods. For instance, Wang et al. [21] reported that a multi-step leaching protocol (i.e., the first and second steps were water leaching, and the third step was lactic acid fermentation broth leaching) achieved 98.7% Cl removal. In this regard, removing inorganic salts via acid-based solutions has been widely applied because it can be affected by the solubility of salts and alkali substances.

Previous studies on water washing of IFA focused on the effects of various experimental parameters such as the liquid-to-solid ratio, washing retention time, and the frequency on Cl removal efficiency [22]. However, washing consumes a large amount of solution and produces large amounts of high-salinity wastewater, resulting in post-processing issues and increased costs of treatment. Therefore, IFA requires suitable pre-treatment to avoid secondary pollution and low-cost treatment. From the perspective of field applications, developing efficient pre-treatment is necessary for inorganic salt removal in IFA, which could not only increase the cost efficiency, but also save resources and energy. A washing ejector based on hydrodynamic cavitation is a pre-treatment technology used to disperse aggregates by cavitation flow. Generally, hydrodynamic cavitation as a phenomenon can be easily induced by low local static pressure in fluid fields [23]. Hydrodynamic cavitation is affected by the Bernoulli principle, and the liquid pressure decreases with the increase in kinetic energy. The Bernoulli equation is as follows:

(1) $$P_{1}+\frac{\rho_{1}V_{1}^{2}}{2}=P_{2}+\frac{\rho_{2}V_{2}^{2}}{2}$$

where P_1_ and P_2_ are the pressures upstream and downstream, and ρ_1_ and ρ_2_ are the densities of the liquid upstream and downstream, respectively. V_1_ and V_2_ are the liquid flow velocities upstream and downstream, respectively. It has been reported that the geometry of the Venturi tube causes the cavitation phenomena in water flow [24,25]. Therefore, the fluid behavior in the washing ejector is governed by the pressure according to the influences of geometrical parameters such as the throat diameter and tube diameter [26]. Based on the feature of cavitation, the device developed in this study utilizes a large number of the bubbles to remove inorganic salts in the IFA. At the same time, the bubbles are mainly attributed to increasing the chemical reaction rate during the cavitation of water. The cavitation behavior is mainly attributed to the enhancement of the hydroxyl radical production as a result of an increased the gas–liquid mass transfer effect [27]. Depending on the flow conditions in the washing ejector, the cavitation flow enhances the mass transfer rate and promotes the dissolution of gas in water.

In our previous work, a washing ejector technology was used in fine particles enriched in contaminated soil with smelting-related contaminants, and it achieved remarkable results [28,29]. It is well known that the accumulation of metals is significantly affected by the contents of organic matter, clay minerals, and Fe oxides in contaminated soil. Thus, before the treatment of the contaminants, enhanced physical separation of fine particles is necessary, along with surface cleaning of the particles from the contaminated soil. Therefore, it is technically feasible to remove contaminated soil containing organic matter via a washing ejector. When using a washing ejector equipment, the volume of the contaminants can be greatly reduced in a shorter time, which is economically desirable.

In general, the effluent produced by the washing process of IFA contains a high Ca content [30]. High-Ca-content effluent in the washing process will form scale in the washing ejector of in the circulation system, causing a blockage of the tube. Given that the effluent contains a high Ca concentration, the present study investigated the influences of the effluent after washing on carbonation. The Ca^2+^ ions from a washing effluent can be removed by precipitation. It is well known that the formation of CaCO_3_ is accelerated under alkaline conditions. In this regard, reducing the amount of washing effluent is expected from the application of CO_2_ capture. This study utilized a washing ejector to remove inorganic salts from the IFA, investigating the effects of washing variables including fluid pressure and the water-to-solid ratio on the residue characteristics. The feasibility of the application of CO_2_ capture and the reuse of the effluent was also investigated using the washing ejector.

## 2. Materials and Methods

### 2.1. Properties of Municipal Solid Waste IFA

The IFA used in this study was obtained from the Seoul Resource Recovery Facility (a local waste-to-energy incineration plant), Korea. The incineration plant is a burn facility that treats 900 tons of solid waste daily. The IFA particles were irregular, exhibited flocculent morphologies, and were colored gray. The average particle size of the IFA was determined using a particle-size analyzer. The median particle size (D_50_) of the IFA was 51.7 µm. The minerals found by the XRD analysis of IFA were CaClOH, CaCO_3_, KCl, and NaCl. Calcite (CaCO_3_) was attributed to the carbonation reaction, which occurred during the IFA storage [30]. The inorganic salts are formed by incinerated plastics, such as polyvinyl chloride and food waste, and CaClOH is formed by the interaction of Ca-based phases with HCl [31]. To investigate the chemical composition of the IFA, X-ray fluorescence (XRF) was performed. It appeared that Ca, Cl, Na, and K were the main elements in IFA, with a Ca and Cl content of 31.2% and 22.9%, respectively. The total concentrations of trace metals in the IFA were extracted using HCl and HNO_3_ at a 3:1 ratio (i.e., aqua regia). The results revealed that Pb and Zn were the most abundant trace metals in the IFA (Table 1).

### 2.2. Experimental Equipment and Conditions

#### 2.2.1. Washing Ejector

**Figure 1:**
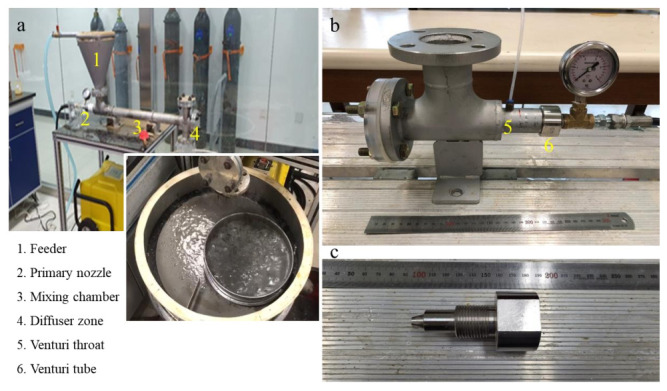
(**a**) Photograph of washing ejector; (**b**) photograph of the cavitating device; (**c**) photograph of nozzle.

A washing ejector is a pre-treatment technology used to disperse IFA aggregates and release contaminants by the collapse of cavitation bubbles. The washing ejector developed in this study is a device that utilizes the cavitation bubbles from the pressure variations in the liquid to remove inorganic salts. In these experiments, tap water was used as the fluid. Figure 1a shows a photograph of the washing equipment on a small scale. The equipment parts were all made of stainless steel. Figure 1b–c show photo details of the cavitating device and a nozzle. A nozzle was attached to the cavitating device, which was connected to a washing ejector. The diameter of the Venturi tube (D) was 5 mm, and the throat diameter (d) was 3 mm with a diameter ratio of 2.78. The washing ejector comprised a feeder, a primary nozzle, a mixing chamber, and a diffuser zone. The feeder was set at the top of the chamber, and the nozzle was parallel to the axis of the ejector. The cavitation bubble was generated by a primary nozzle in the water. The cavitating flow was sprayed from the nozzle into the mixing chamber zone, where the IFA was completely mixed via the feeder. The mass flow was discharged to a diffuser zone, placed at the end of the ejector. In particular, Cl-related phases in the IFA are considered to be water soluble and tend to deposit on the surface of particles; thus, they are easily removed by a washing ejector. Therefore, the collision of cavitation bubbles can occur on the particle surfaces, weakly binding to the IFA surface.

**Figure 2:**
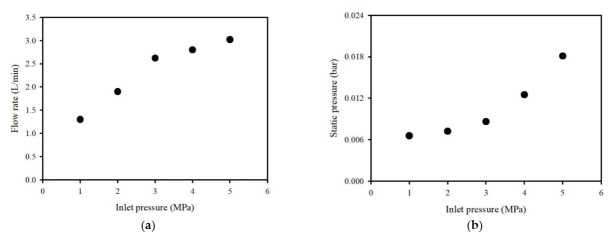
Influence of (**a**) flow rate and (**b**) static pressure drop as a function of inlet pressure using a washing ejector.

In order to evaluate the cavitation flow using the washing ejector, the inlet pressure was controlled using the flow control valves, ranging from 1 to 5 MPa gauge pressure. Figure 2 presents the flow rate and static pressure as a function of inlet pressure. The flow rate was 1.3 L/min for inlet pressure of 1 MPa, 2.6 L/min for 3 MPa, and 3.0 L/min for 5 MPa. With the increase in the inlet pressure, the static pressure difference increased from 0.007 bar to 0.01 bar. Eventually, the static pressure affected the cavitation flow in the washing ejector.

Washing experiments were conducted to assess the efficiency of the washing process to remove the inorganic salts in the IFA. Three ratios (water-to-solid), i.e., 1:1, 1.5:1, and 2:1, were investigated in this study, and the fluid pressures were selected as 1 MPa, 3 MPa, and 5 MPa, respectively. All experiments were duplicated. The major components of the IFA and the washed IFA were analyzed by X-ray fluorescence spectroscopy (XRF). To investigate the impacts of cavitating flow on washing treatment using a washing ejector, the residues were recovered and compared with the IFA via XRD and Fourier transform infrared spectroscopy (FTIR).

#### 2.2.2. Characteristics of the Washed IFA Residue

The thermogravimetric behavior of the IFA, before and after treatment, was analyzed using a thermogravimetric analyzer (TGA) under N_2_ and air environments. The SEM measurement was performed to characterize the washed residue.

IFA has been reported to contain various toxic metals. To determine the potential release of toxic elements from samples, the Korean standard leaching test (KSLT) was conducted using the modified method [32]. The modified KSLT was performed to analyze the release characteristics of the toxic elements in the samples at various pH values. Briefly, 50 g of sample was added to 500 mL of water at various initial pH values from 1–9 and agitated at 200 rpm for 24 h. The concentrations of toxic elements in the extracts were analyzed using ICP. In the IFA, the concentrations of heavy metals were under the regulation level in Korea. These results indicate a low possibility of secondary contamination.

The concentrations of dissolved Cl and other components that did not form solid phases were measured using ion chromatography (IC) and inductively coupled plasma-optical emission spectrometry (ICP-OES), respectively.

### 2.3. Carbonation Reaction for the CO_2_ Capture from the Washing Effluent

The experiments for carbonation involving the reuse of the washing effluent and CO_2_ capture proceeded as follows. After washing, the residues were separated by filtration, and pure CO_2_ was then continuously injected through a gas disperser (at a flow rate of 1.5 L/min and 3.0 L/min) into the washing effluent (3 L). An open-glass reactor was used, without stirring. The CO_2_ was injected at a fast flow rate to accelerate the carbonation reaction because pure CO_2_ is much more concentrated than CO_2_ in the atmosphere. In our experiments, when the pH of the solution reached approximately 8.0, the CO_2_ injection was stopped. The carbonation efficiency was calculated based on the difference in Ca concentration before and after the injection of CO_2_. After the end of carbonation, the precipitate was analyzed using XRD.

### 2.4. Analytical Methods

The thermogravimetric behavior of the sample was analyzed using TGA (SDT Q600, TA Instruments, New Castle, DE, USA). The thermogravimetric behavior of the sample was analyzed at a heating rate of 10 °C/min under N_2_ and air; the scan range was approximately 1100 °C. The sample was analyzed via XRD (X’Pert Pro MRD, Malvern PANalytical, Almelo, The Netherlands). Cu–Kα X-rays were used at an acceleration voltage of 40 kV, with a current of 30 mA. The sample was analyzed for 2θ values from 10–70° to determine the mineral phase composition. The elemental composition of the samples was determined by XRF spectrometry (S4 PIONEER, Bruker AXS, Karlsruhe, Germany). Fourier transform infrared spectroscopy (FTIR, Nicolet 6700, Thermo Fisher Scientific, Waltham, MA, USA) was used to obtain the infrared spectra. The surface morphologies of the samples were analyzed via FE-SEM (S4800, Hitachi, Tokyo, Japan) with EDS. The average particle sizes of the samples were determined using a particle size analyzer (Mastersizer 2000, Malvern Panalytical Ltd., Malvern, UK). After washing, the effluent was collected and filtered through a 0.45 μm membrane filter. The concentration of effluent in the filtrate was measured using IC (883 Basic IC Plus, Metrohm, Runcorn, UK) and ICP-OES (Perkin Elmer Optima Model 5300DV, PerkinElmer Inc., Waltham, MA, USA), respectively. The pH and EC were determined using a multi-parameter probe (U-50, HORIBA, Advanced Techno Co., Ltd., Fukuoka, Japan).

## 3. Results

### 3.1. Removal of Inorganic Salts of the IFA using a Washing Ejector

The major elements of IFA and washed IFA are listed in Table 2. The fluid pressure and water-to-solid ratio influence the removal of inorganic salts during water washing. The influence of fluid pressure on Cl removal was studied at a water-to-solid ratio of 1:1. The Cl content in the IFA was 5.01%, 3.47%, and 3.06 % for fluid pressures of 1 MPa, 3 MPa, and 5 MPa, respectively. Other insoluble components, such as Al_2_O_3_ and SiO_2_, could not be removed by washing; therefore, their proportions increased due to the removal of inorganic salts from the residue. In addition, in the experiments using a washing ejector, we observed that when the fluid pressure was 5 MPa, the Cl content in the residue after washing was 3.06 %, 2.72 %, and 1.52 %, respectively, for a water-to-solid ratio from 1 to 2. Thus, when washing ejectors are used to remove inorganic salts from IFA, the contribution of dissolution to the removal of inorganic salts is even higher at fluid pressure, and a larger water-to-solid ratio can promote contact between the solid and water.

**Figure 3:**
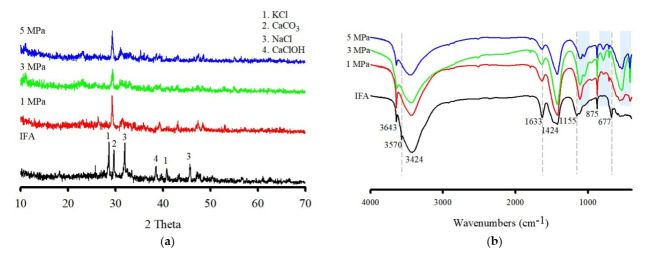
The XRD patterns (**a**) and FTIR (**b**) of IFA before and after using a washing ejector.

The variations in the mineral composition of the IFA were evaluated via XRD analysis (Figure 3a). The crystalline phases were calcite (CaCO_3_), halite (NaCl), sylvite (KCl), and CaClOH, which also contain amorphous phases. After washing with IFA, the characteristic peaks of KCl, NaCl, and CaClOH disappeared, and the peak intensity of calcite showed no change and no formation of new peaks through Ca dissolution. In this regard, it could be inferred that the newly formed precipitate from the washing reaction was amorphous CaCO_3_. Considering the XRD analysis, no changes in the amorphous components were identified. Therefore, we studied the changes in Ca- and Cl-related functional groups during washing ejector treatment, and FTIR analyses were conducted to confirm the changes (Figure 3b).

The spectrum of the original IFA exhibited bands at 3643 cm^−1^, 3570 cm^−1^, and 3424 cm^−1^ (O–H stretching). The bands at 3643 cm^−1^ and 3570 cm^−1^ are related to the stretching vibration of Ca-OH in Ca(OH)_2_ and the bending vibration of CaClOH. In addition, the band at 1633 cm^−1^ is associated with the vibration of the H-O-H band in the interlayer water [33]. The bands at 1424 cm^−1^ and 875 cm^−1^ are attributed to the presence of amorphous CaCO_3_ and calcite, respectively [34]. The bands around 1155 cm^−1^ and 677 cm^−1^ are related to the presence of sulfate [35]. After washing with IFA, the bands at 3643 cm^−1^ and 1633 cm^−1^ gradually decreased with the increasing fluid pressure, while the band at 1424 cm^−1^ increased and another band at 712 cm^−1^ appeared, corresponding to calcite. This result indicated that Ca(OH)_2_ gradually participated in the washing reaction and that much Ca(OH)_2_ transformed into amorphous CaCO_3_ in proportion to the increase in fluid pressure. Subsequent interactions with dissolved CO_2_ led to the formation of amorphous CaCO_3_. In contrast, the bands at 3570 cm^−1^, 1155 cm^−1^, and 677 cm^−1^ disappeared, which may be due to the solubility of the compounds. Simultaneously, the bands appearing at approximately 1100 cm^−1^ and 1040 cm^−1^ are related to the stretching asymmetric vibrations of Si, and the absorption peaks at approximately 786 cm^−1^, 532 cm^−1^, and 457 cm^−1^ are assigned to the bending vibrations of Si-O groups.

### 3.2. Characteristics of the Effluent after Washing

According to the above results, the inorganic salts in IFA were easily washed away. Table 3 presents the release of high concentrations of Cl, SO_4_, and dissolved solids. In addition, Ca, K, and Na were detected at high concentrations in the effluent. It can be seen that the effluent after washing had a high pH and conductivity due to the solubility of hydroxides and inorganic salts, which could be caused by the high CaO and Cl content of IFA. After washing, the total content of inorganic salt elements, such as Cl, Na, and K, in the washed residue decreased to approximately 90%, in which the contents of Cl, Na, and K decreased from 22.9 to 1.52%, 7.66 to 0.91%, and 4.31 to 0.68%, respectively (Table 2). Therefore, the compound dissolves according to Equations (2)–(4).

During the washing process, HCO_3_ can be formed while the cavitation flow in the washing ejector process reacts with CO_2_ in ambient air (Equations (5)–(6)). Theoretically, CO_2_ dissolution in water follows Henry’s law, with the dissolved amount directly proportional to the partial pressure of CO_2_ in air. As such, HCO_3_ can react with free Ca in the solution to form amorphous CaCO_3_; this is because there were sufficient Ca compounds in the particles so that inorganic salts in the surface dissolved into the solution, followed by a reaction with the free Ca or Ca compounds. Therefore, by removing inorganic salts, the surface of the washed IFA would be affected by the newly formed precipitate from the reaction (Equations (7)–(9)). In addition, high pH has more reducing conditions in the effluent, and release elements can mainly exist in the reducing form. In this regard, carbonates and a variety of complexes were formed during washing (Equation (10)), indicating that the compound was soluble in water.

(2) $$\mathrm{NaCl}\left(s\right)↔\mathrm{NaCl}\left(\mathrm{aq}\right)$$

(3) $$\mathrm{KCl}\left(s\right)↔\mathrm{KCl}\left(\mathrm{aq}\right)$$

(4) $$2\mathrm{CaClOH}\left(s\right)↔\mathrm{Ca}{\left(\mathrm{OH}\right)}_{2}\left(s\right)+{\mathrm{CaCl}}_{2}\left(\mathrm{aq}\right)$$

(5) $$H_{2}O+{\mathrm{CO}}_{2}↔H_{2}{\mathrm{CO}}_{3}\;$$

(6) $$H_{2}{\mathrm{CO}}_{3}↔H^{+}+{\mathrm{HCO}}_{3}^{-}$$

(7) $$\mathrm{CaO}+H_{2}O↔\mathrm{Ca}{\left(\mathrm{OH}\right)}_{2}\;$$

(8) $$\mathrm{Ca}{\left(\mathrm{OH}\right)}_{2}↔{\mathrm{Ca}}^{2+}+2{\mathrm{OH}}^{-}\;$$

(9) $${\mathrm{Ca}}^{2+}+{\mathrm{CO}}_{3}^{2-}↔{\mathrm{CaCO}}_{3}$$

(10) $${\mathrm{nCO}}_{2}+2M\left(\mathrm{Metal}\right)+{\mathrm{nH}}_{2}O↔{\left(M^{n+}\right)}_{2}{\left({\mathrm{CO}}_{3}^{2-}\right)}_{n}+2{\mathrm{nH}}^{+}$$

### 3.3. Characteristics of the Washed IFA Residue

#### 3.3.1. TGA

**Figure 4:**
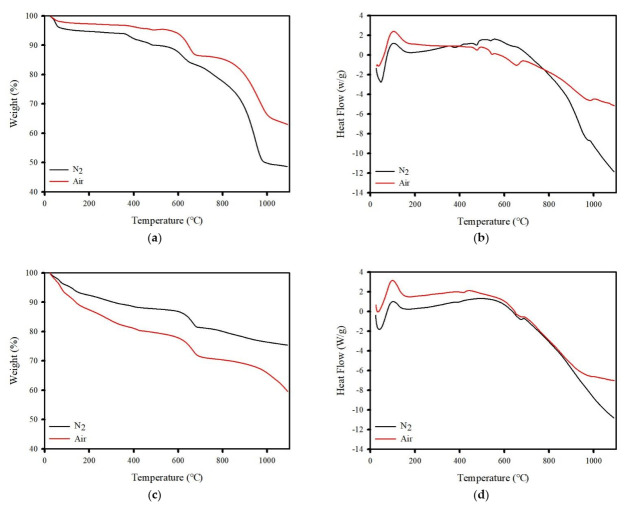
The TG/DTA of IFA (**a**,**b**) and washed IFA (**c**,**d**) using a washing ejector (at L/S 2 and 5 MPa).

The TG and DSC curves of the IFA at a heating rate of 10 °C/min under an inert atmosphere of N_2_ and air are shown in Figure 4. The mass loss of IFA and washed IFA showed different trends with increasing temperature. TGA was conducted under N_2_ conditions to characterize the thermal behavior of the IFA, and a stage of sharp mass loss above 820 °C was found in the high-temperature region. This peak accounts for approximately 27.8% of the mass loss, whereas that of the washed IFA above 820 °C was 4.21%. The difference in the mass loss variation may have been caused by inorganic salts. Additionally, at temperatures above 650 °C in the N_2_ environment, the mass loss is mainly attributed to the thermal decomposition of CaCO_3_ in the samples and the formation of CaO when CO_2_ is released (Equation (11)). However, the mass loss variation of the samples in the air conditions showed different trends with increasing temperatures, as compared to N_2_. Based on the TG curves, the washed IFA mass significantly decreased until 600 °C, which can be attributed to the endothermic reaction. This phenomenon may be caused by the amorphous compound and decomposition of Ca(OH)_2_, i.e., Ca(OH)_2_ decomposed because it was present in the washed IFA, in agreement with the FTIR results.

(11) $$CaCO_{3}\to \mathrm{CaO}+CO_{2}$$

#### 3.3.2. The pH Leaching Characteristics

**Figure 5:**
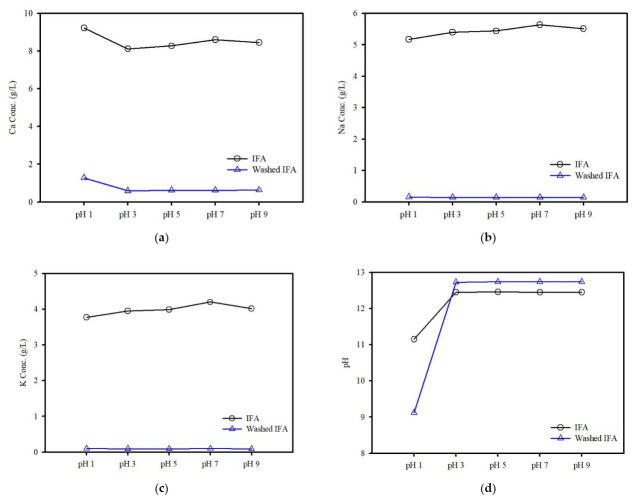
(**a**–**c**) Effect of the initial pH on the Ca, Na and K concentration leached from the IFA and washed IFA and (**d**) leachate pH at different initial pH.

Considering the influence of the washing treatment on Ca content, the pH could significantly affect the solubility of hydroxides. After washing, the relative content of Ca increased from 31.2 to 37.7% (Table 2). However, a potentially soluble compound can be affected by the solubility of IFA. Therefore, the IFA and washed IFA under different pH conditions using the modified KSLT were analyzed to understand the element release (Figure 5). Generally, the leachate pH significantly influences the leaching behavior of metals; however, it was below the detection limits for all pH values used in this study (<0.1 mg/L). The results of the present study revealed that the leaching of Ca for the IFA did not influence the response for all pH ranges, except at a pH of 1.0. The Ca concentration for the washed IFA in all experimental conditions was lower than the concentration in the IFA and the leaching behavior of Ca hardly changed before and after washing. In contrast, all the leachate for the various pH conditions increased from the initial pH values. This indicated that the soluble Ca compound acted as a pH buffer because of the excessive amount of Ca dissolution in the leachate.

#### 3.3.3. SEM

**Figure 6:**
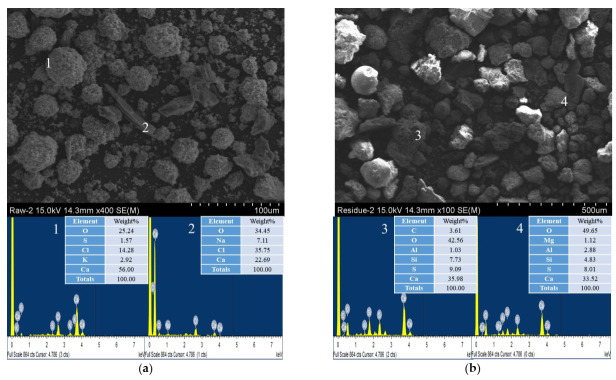
The SEM images and the corresponding EDS analysis of IFA before (**a**) and after (**b**) using the washing ejector.

SEM images of the IFA and washed IFA are shown in Figure 6. The IFA had an irregular shape, consisting of uneven surface spherical and rod crystals. The IFA particles showed that the spherical crystals were characterized as mixtures of Ca minerals and were mixed with irregular rod crystals. The EDS spectrum analysis revealed that the spherical crystals were composed of grains surrounded by soluble salts and contained large amounts of Ca, Cl, and O. These metal chloride forms were unstable and easily solubilized in water. After washing treatment, the surface of the particles was relatively smooth, the amount of soluble salt decreased, and the trace elements increased somewhat. Therefore, the cavitation bubble influences the particles and can produce clean IFA surfaces.

## 4. Discussion

### 4.1. Carbonation for CO_2_ Capture on the Washing Effluent

**Figure 7:**
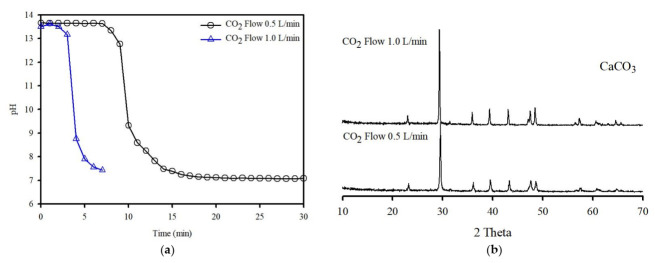
(**a**) Changes in pH in the effluent at various CO_2_ flow rates. (**b**) XRD patterns of the precipitates obtained under CO_2_ flow rates.

The washing effluent plays a major role in sequestration, providing an abundant quantity of Ca, and may be sufficiently reactive to be used as a medium for the formation of CaCO_3_. In this study, effluent carbonation was conducted to investigate the effects of the injection flow rate of CO_2_ on the precipitation process; the results are shown in Figure 7a. The CO_2_ injection was stopped at approximately pH 8.0, and the effluent attained a steady pH value. When CO_2_ was injected into the effluent, the pH decreased very quickly in the pH range of approximately 13.0–9.0; this indicates the completion of the carbonation reaction within this pH range. The form of CO_2_ is dependent on the pH of the aqueous phase, where the dominant species is CO_3_ [36]. When CO_2_ was taken into the effluent, the effluent lost its ability to buffer the pH, and the Ca then approached equilibrium.

The carbonation reaction can be accelerated by the consumption of hydroxide ions caused by alkaline conditions. Therefore, more CO_2_ could be dissolved in the washing effluent as the CO_2_ flow rate increased, resulting in a rapid decrease in pH. After the end of carbonation, carbonation efficiencies of 52.0% (4 min) and 53.3% (10 min) were obtained with 1.5 L/min and 3.0 L/min, respectively. It has been shown that carbonation efficiency is relatively restricted in the chemical composition of this effluent. The effectiveness of carbonation efficiency in solution depends on the temperature, pressure, and chemical composition, and CO_2_ dissolution can react with the soluble compound in many ways [37]. A considerable amount of soluble salts was dissolved from the IFA, which enabled the precipitation process to occur at the same time. The presence of high amounts of soluble salts has been confirmed by Visual MINTEQ to have high ionic strength, and, thus, the presence of soluble salts may hinder the CO_2_ absorption process. These results are in agreement with those of other studies [38,39]. Therefore, the CO_2_ flow rate has a distinct effect on carbonation efficiency, and the ionic strength has a major effect on carbonation efficiency. The XRD patterns of the precipitates obtained after carbonation are shown in Figure 7b; as can be seen, the precipitate was composed of calcite, and the calcite peaks increased in intensity after carbonation under different flow rate conditions.

### 4.2. Effect on the IFA by Cavitation Bubbles and Prospects

**Figure 8:**
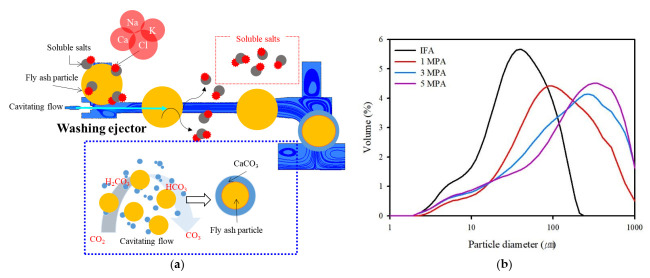
(**a**) Diagram of the mechanism of the phase changes of IFA after using the washing ejector. (**b**) Particle size distribution before and after using the washing ejector.

The primary objective of the pre-treatment was to determine the effect of removing inorganic salts in IFA using the washing ejector, followed by the prospect of a sustainable method of utilizing IFA. In the hydrodynamic cavitation using a washing ejector, a large number of water molecules diffused into the cavitation bubble. When the liquid was then subjected to a higher pressure, these bubbles collapsed, which can be attributed to the chemical reaction. The removal mechanism of the inorganic salts using a washing ejector can be summarized from the above results (Figure 8a). The inorganic salt-rich layer from the IFA can easily dissolve in cavitation bubbles. The soluble compound can be affected by collapse due to cavitation bubble formation during the washing process, and Ca release was accelerated to form aqueous CaCO_3_, which reacted with carbonates to generate precipitates under aqueous conditions. The main reason for this phenomenon is that the carbonates in the aqueous solution reacted with the released Ca ions to be deposited on the surface of the particles, indicating that the amount of amorphous CaCO_3_ gradually increased during washing (Equations (6)–(8)). These carbonates accumulated to form amorphous CaCO_3_, hindering the further dissolution of soluble compounds or trace elements, such as heavy metals, resulting in the inability to continue dissolution. Amorphous CaCO_3_ can be produced in the reaction, which can be explained by the particle distribution. The particle size distribution of the washed IFA was measured using a laser particle-size analyzer. As shown in Figure 8b, the median particle sizes (D_50_) were 108 µm, 161 µm, and 192 µm for fluid pressures of 1 MPa, 3 MPa, and 5 MPa, respectively. The washing ejector was accompanied by an increase in amorphous CaCO_3_, as well as a decrease in chloride content.

Many studies have investigated the composition of incineration residues and these have good potential for reuse and recycling owing to their suitable physical, chemical, and mineralogical properties. Despite being a beneficial industrial byproduct for use in various applications, the hazardous composition in the IFA is the key factor that restricts its comprehensive utilization. The removal of hazardous components such as MSW-IFA is very important for the enhancement of recycling, as it helps to drive the concept of sustainable development for material recovery (to perform disposal and utilization methods). In this study, the effects of the generation of cavitation bubbles for the removal of inorganic salts in IFA were studied via a washing ejector. Hydrodynamic cavitation has chemical and physical attributes, such as the formation of micro- or nanobubbles and the generation of free radicals. Although cavitation bubbles are attracting significant attention due to their unique physiochemical characteristics such as the improved mass transfer at the gas–liquid interfaces, the use of hydrodynamic cavitation technologies has gained significant attention in various applications (e.g., mineral processing, chemical reactions, and water purification) because of their superior characteristics. Therefore, the application of hydrodynamic cavitation is possible for the remediation of contaminated sediments, MSW-IFA, and soils by removing hazardous chemicals while reducing energy requirements. Overall, there is a need to promote a comprehensive understanding of hydrodynamic cavitation. In order to determine the efficiency of field operations, great attention should be paid to developing sustainable recycling methods for IFA.

## 5. Conclusions

In this study, we used a washing ejector to remove inorganic salts from the IFA. Particle–bubble collisions were accomplished through cavitation bubble formation; thus, the soluble salt removal efficiency was enhanced. The washing processes for inorganic salt removal in IFA under different conditions were studied, employing as little water as possible (i.e., at a liquid-to-solid ratio of 2:1). We confirmed that the change in the inlet pressure has a significant effect on the removal efficiency of inorganic salts in the IFA. It was found that the removal efficiency of inorganic salts in IFA is related to the cavitation bubbles in the solution, which influence cavitation behavior. When cavitation bubbles infiltrate through the surface IFA, inorganic salts and Ca compounds dissolve into the cavitating flow. This study discussed the influence of a washing ejector on the removal of soluble salts by IFA and its release mechanism. The main conclusions are as follows:

1. When a washing ejector is used for the removal of inorganic salts from the IFA, the contribution to the removal of inorganic salts is even high at fluid pressure, and a larger water-to-solid ratio can promote contact between the solids and water.
2. The cavitation bubble influences the particles, and it can produce clean IFA surfaces. In addition, HCO_3_ can be formed while the cavitation flow in the washing ejector process reacts with CO_2_ in ambient air. As such, HCO_3_ can react with free Ca in the solution to form amorphous CaCO_3_.
3. The washing effluent plays a major role in CO_2_ sequestration, thereby providing an abundant quantity of Ca and may be sufficiently reactive for use as a medium in the formation of CaCO_3_.

During washing, the release is achieved through the dissolution of inorganic salts, such as soluble compounds weakly bound to the surface. Hence, the cavitation flow via the Venturi tube of the ejector can remove the surface Ca and Cl-related functional groups, along with surface cleaning of the particles. Removing the inorganic salts in the IFA altered the characteristics of the Ca-related phase, and amorphous CaCO_3_ was formed. According to the results, the release of Ca and Cl in the IFA could cause carbonation, and these tendencies were enhanced as the bubbles increased owing to the diffusion caused by the mass transfer.

## Figures and Tables

**Table 1:** XRF (wt. %) and ICP (* mg/kg) analysis results for the IFA.

Ca	Cl	Na	K	Si	S	Mg
31.2	22.9	7.6	4.3	2.1	2.0	1.1
Pb *	Cd *	As *	Cu *	Zn *	Cr *	Fe *
9313.3	1795.5	167.9	8213.3	88,609.2	2456.6	204,518.4

**Table 2:** Major elements of various parameters in IFA and washed IFA samples (wt. %).

Composition	Untreated	Water-to-Solid Ratio				
	IFA	1:1	1:1	1:1	1.5:1	2:1
		1 MPa	3 MPa	5 MPa	5 MPa	5 MPa
Cl	22.9	5.01	3.47	3.06	2.72	1.52
Na_2_O	10.3	2.03	1.93	1.54	1.37	1.22
K_2_O	5.20	1.68	1.23	1.13	1.01	0.81
CaO	43.6	57.7	52.1	51.6	53.3	52.7
SiO_2_	4.55	8.66	8.68	9.1	8.96	9.80
Al_2_O_3_	1.79	4.24	9.85	9.9	10.5	10.1
MgO	1.82	3.91	3.14	3.0	3.31	3.18
Fe_2_O_3_	1.34	2.85	4.62	5.9	4.45	6.86
TiO_2_	1.31	3.31	4.37	4.5	4.39	4.64
SO_3_	5.02	6.67	6.16	5.7	5.83	5.61

**Table 3:** Characteristics of effluent from washing ejector.

pH	EC (S/m)	Cl (g/L)	Br (g/L)	SO_4_ (g/L)	Ca (g/L)	Na (g/L)	K (g/L)
13.8	3.3	19.84	0.45	1.29	5.24	3.10	2.92

## Data Availability

All data generated or analyzed during this study are included in this published article.
